# Experiments Relative to the Controversy between Dr. Wilson Philip and Mr. Brodie

**Published:** 1820-05

**Authors:** Clarke Abel

**Affiliations:** one of the Physicians to the Brighton Dispensary and Sussex General Infirmary.


ORIGINAL COMMUNICATIONS, continued from page 379.

FOR THE LONDON MEDICAL AND PHYSICAL JOURNAL.

### *Experiments relative to the Controversy between Dr. Wilson Philip and Mr. Brodie.* By Clarke Abel, M.D. F.R.S. one of the Physicians to the Brighton Dispensary and Sussex General Infirmary.*

THE following experiments were made in reference to the question at issue between Dr. Wilson Philip and Mr. Brodie; namely, whether galvanism, when applied to the thoracic and

* This paper is separated from the rest of our *Original Communications,* in consequence of its having been received after the foregoing part of the Journal had passed the press.—Edit.

gastric portions of the eighth pair of nerves, after their division, can produce the appearances of digestion. They are repetitions of experiments performed by those gentlemen.

*Experiment 1st.*—Two rabbits of equal size, and about five months old, were used for the first experiment. After a fast of eighteen hours, they were allowed to eat as much parsley as they chose. As soon as they had ceased to feed, the par vagum was divided on each side of the neck of the animal to which the galvanism was not to be applied. An hour and a half afterwards, the nerves of the other rabbit were divided, and their lower ends inclosed in two pieces of tin-foil, which were brought into contact. The hair over the region of the stomach was shaved off to the extent of four square inches, and a folded piece of tin-foil, inclosing a half-crown piece, was firmly bound on the denuded part. The animal was then brought under the influence of a galvanic battery, by connecting the positive wire with the tin-foil including the nerves, and the negative wire with the tin-foil covering the region of the stomach. The battery consisted of fifty pairs of three-inch plates, and was charged with diluted muriatic acid, in the proportion of one of acid to five of water. Not more than one-third of the plates was employed whilst the battery continued in full action; as this diminished, their number was increased. The power of the battery was also supported by occasional additions of acid. The galvanism was applied in a continued stream. The power kept up during the whole experiment was sufficient to produce strong contractions of the fore legs, whenever the stream was interrupted: so long as it flowed steadily, no motion of the legs was perceptible. The respiration of the animal continued quite free during the experiment, except when the disengagement of the nerves from the tin-foil rendered a short suspension of the galvanism necessary during their re-adjustment. Two efforts to vomit occurred: one, four hours after the division of the nerves, during a short discontinuance of the galvanism; the other, on the sudden application of a greater power, in consequence of a fresh supply of acid to the battery.

After the experiment had lasted eleven hours and a half, the animal was set at liberty. It was at first remarkably lively, but in a few minutes began to breathe with great difficulty. Having been suffered to live half-an-hour, that its respiration might be observed, it was killed by a blow on the occiput.

Whilst the experiment was going on, the non-galvanized rabbit breathed with difficulty, wheezed audibly, and made frequent attempts to vomit. Its distress, indeed, at length became so great, that I was induced to kill it an hour and a half before the termination of the experiment. The nerves of the galvanized rabbit not having been divided till an hour and a half after those of the other, both animals lived equal times after their division.

On opening the chest of the non-galvanized rabbit, the lungs were found covered with dark-coloured blotches. The trachea, and especially its ramifications, contained a large quantity of frothy fluid. The stomach was greatly distended, and, on being laid open from the pylorus to the cardia, emitted a strong smell of parsley. Its contents were of a dark-green colour, and resembled in all respects masticated parsley: no vestige of other food was found in the stomach.

The lungs of the galvanized rabbit had some blotches on their surface; but these were smaller, much less numerous, and less darkly-coloured than those in the lungs of the other animal. The stomach was much distended: its contents very nearly resembled those of the stomach of the non-galvanized rabbit, and they had the smell and colour of parsley.

This experiment proves that galvanism of a certain power, passed in a continued stream through the thoracic and gastric portions of the eighth pair of nerves, after their division, will prevent the accession of dyspnœa and of efforts to vomit; both of which are the consequences of the operation, when no galvanism is applied. It also proves that the same power is incapable of producing the phenomena of digestion. To ascertain if they could be produced through a greater power, a second experiment was performed.

*Experiment 2d.*—The subjects of this experiment were two rabbits three months old, and of equal size. After a fast of eighteen hours, having been fed the morning preceding that of the experiment with oats and bran, they were allowed to eat as much parsley as they chose. As soon as they had ceased to feed, the eighth pair of nerves were divided in both. One of them was allowed to remain at rest. The other, having its nerves and the region of its stomach coated with tin-foil, as in the foregoing experiment, was immediately brought under the influence of galvanism. The entire power of the battery of fifty pairs of three-inch plates, charged with diluted muriatic acid in the proportion of one to five, and kept in full action, was applied in a stream throughout the experiment. This power produced no continued twitching when applied uninterruptedly, but caused a fixed extension of the fore legs. When applied by successive contacts, it occasioned violent contractions of all the muscles of the body. The experiment lasted nine hours, when the rabbit died from the effects of the galvanism. The respiration of the animal was easy during the first five hours of the experiment, but became rather laborious during the last two hours, and especially during the last half-hour. To relieve it, the power of the battery was increased by a fresh addition of acid; but the animal was too much exhausted to bear the increase. Its application produced a universal spasm, and destroyed the animal. No attempts to vomit occurred during the experiment.

The non-galvanized rabbit laboured exceedingly in its breathing, and made frequent efforts to vomit; its uneasiness continually increasing, till the close of the experiment on the galvanized rabbit: it was then destroyed by a blow on the occiput.

The lungs of the non-galvanized rabbit had some livid spots on its surface, but of much smaller extent than those observed in the non-galvanized rabbit of the former experiment. The cavity of the pleura contained much serous fluid. The trachea, and its ramifications, contained a very little frothy mucus. The stomach was much distended: when slit open from the pylorus to the cardia, it disclosed a continuous mass of masticated parsley, of a dark-green colour, and of its natural odour. The œsophagus was full of a similar matter, but in a moister state. No remains whatever of other food were found in the stomach.

The lungs of the galvanized rabbit were highly inflamed, resembling liver, excepting their margins, which were of a light colour. The bronchiæ contained a large quantity of frothy fluid. The stomach was distended, but contracted in its middle: on being slit open, it displayed its contents divided into two parts; one occupying its pyloric, the other its cardiac, division. The cardiac portion of the contents was for the most part of a faded-green colour, which became deeper towards its centre. Near the œsophagus was a part in a semi-fluid state, and of a brown colour. The whole of the cardiac portion of the contents was very moist. The pyloric part of the mass was of a brownish colour, and resembled the contents of a healthy rabbit's stomach, except towards the centre, where it had a greenish maculated appearance, seemingly from the mixture of parsley in different states of digestion. The whole of the contents had a faint smell of parsley, but very different from that of the contents of the stomach of the other rabbit. The œsophagus was quite empty.

From this experiment it seems legitimate to infer, that galvanism of a certain power, passed in a continued stream through the thoracic and gastric portions of the eighth pair of nerves, after their division, will produce the phenomena of digestion. It entirely corresponds, both in its details and results, with the experiments of Dr. Wilson Philip.

Several medical gentlemen did me the favour of witnessing these experiments during their progress, and of examining the contents of the stomach.

*Brighton; April 16th,* 1820.

