# Erratum

**Published:** 1820-07

**Authors:** 


ERRATUM *in the last Volume.*—Page 387, five lines from the bottom, *nine* is printed, instead of *seven*.

